# Necroptosis in Niemann–Pick disease, type C1: a potential therapeutic target

**DOI:** 10.1038/cddis.2016.16

**Published:** 2016-03-17

**Authors:** A Cougnoux, C Cluzeau, S Mitra, R Li, I Williams, K Burkert, X Xu, C A Wassif, W Zheng, F D Porter

**Affiliations:** 1 Program in Developmental Endocrinology and Genetics, Eunice Kennedy Shriver National Institute of Child Health and Human Development, National Institutes of Health, DHHS, Bethesda, MD 20892, USA; 2 Laboratory of Molecular Immunology and the Immunology Center, National Heart, Lung, and Blood Institute, National Institutes of Health, DHHS, Bethesda, MD 20892, USA; 3 National Center for Advancing Translational Sciences, National Institutes of Health, DHHS, Bethesda, MD 20892, USA

## Abstract

Niemann–Pick disease, type C1 (NPC1) is a neurodegenerative, lysosomal storage disorder due to mutation of the *NPC1* gene. The NPC1 phenotype is characterized by progressive neuronal dysfunction, including cerebellar ataxia and dementia. There is histological evidence of neuroinflammation and progressive neuronal loss, with cerebellar Purkinje cells particularly vulnerable to loss of NPC1 function. Necroptosis was evaluated as a mechanism of neuronal loss. Receptor-interacting protein kinase 1 (RIP1) and RIP3 are key components of the necrosomal complex that regulates necroptotic cell death. We report increased expression of RIP1 and RIP3 in NPC1 fibroblasts, NPC1 iPS cell-derived neuronal precursors, and in cerebellar tissue from both NPC1 mice and patients. Our data suggest a positive correlation between NPC1 neurological disease severity and assembly of the necrosome complex. Furthermore, we demonstrate that pharmacological inhibition of RIP1 decreases cell death both *in vitro* and *in vivo*. Treatment of *Npc1*-mutant mice with necrostatin-1, an allosteric inhibitor of RIP1, significantly delayed cerebellar Purkinje cell loss, progression of neurological symptoms, and death. Collectively, our data identified necroptosis as a key component of the molecular network that contributes to neuronal loss in NPC1 and establish that inhibition of necroptosis is a potential therapeutic intervention.

Niemann–Pick disease, type C (NPC) is a rare, inherited, neurovisceral, lysosomal storage disorder. The incidence of classical NPC has been estimated to be on the order of 1/100,000, while there may be a more frequent late-onset variant.^1, 2^ NPC can be caused by mutation in either *NPC1* or *NPC2*, with the majority of cases (~95%) being due to mutation of *NPC1*.^1^ Impaired function of either NPC1 or NPC2 results in the endolysomal storage of unesterified cholesterol and other lipids.^1, 3, 4^ The NPC1 phenotype is heterogeneous with respect to both age of onset (infantile, childhood, adolescent, adult) and disease manifestations. Neurological manifestations are typically associated with progressive cognitive impairment and cerebellar ataxia. Progressive neuronal loss, in particular the long-distance projection neurons of the cerebellar cortex, the Purkinje cells, underlies many of the pathological features observed in NPC1. Miglustat (a glycosphingolipid synthesis inhibitor) has been approved in the European Union and multiple other countries for the treatment of NPC1, it has not been approved by the FDA and its impact on disease progression is limited.^5, 6^

Very little is known about the cellular death mechanisms leading to neuronal loss in NPC1, and thus the potential efficacy of cell death inhibitors remains unexplored. Recent *in vitro* studies have demonstrated defective autophagy in cellular models of NPC1 (fibroblasts and induced pluripotent stem (iPS) cell-derived neurons)^7, 8^ and mouse models of NCP1 disease.^7, 9^ Increased DNA damage demonstrated by both terminal deoxynucleotidyl transferase dUTP nick end labelling staining and electron microscopy, as well as increased expression of genes associated with death receptor signalling have been described in brain tissue from *Npc1*-mutant mice.^10^ Consistent with the understanding of programmed cellular death at that time, it was concluded that neuronal death was due to apoptosis.^11^ Based on this observation, Erickson and Bernard^11^ evaluated the therapeutic potential of inhibiting caspase-dependent cell death using minocycline and transgenic expression of Bcl-2. Neither approach was effective in delaying the onset of neurological signs nor increasing lifespan in *Npc1*^−/−^ mice, thus this group concluded that neuronal loss in NPC1 is not due to apoptosis. Minocycline is a caspase 1 inhibitor,^11^ and thus in retrospect, these data argue against pyroptosis, rather than apoptosis, as the mechanism of cell death in NPC1.^12^ Although caspase-mediated apoptosis is the most commonly investigated mechanism of programmed cell death, several other types of programmed cell death can occur.^13, 14, 15^ Specifically, in the absence of caspase involvement, the most common pathway is necroptosis.^13, 14, 15, 16^ In fact, Erikson and Bernard^11^ noted that prior pathological studies^17, 18^ suggested that neuronal loss in NPC1 might be due to necrosis rather than apoptosis. The inconclusive findings involving inhibition of apoptosis (Bcl-2 transgenic) and pyroptosis (minocycline), along with the pathological studies suggesting ‘necrosis', led us to investigate the possible involvement of necroptosis in NPC1.

Although descriptions of caspase-independent programmed cell death were previously described,^19^ necroptosis was initially delineated by Degterev *et al.*^20^ Necroptosis is mediated by a heterologous protein complex known as the necrosome, which is a multiprotein complex, which includes the two protein kinases, receptor interacting protein kinase 1 (RIP1) and receptor interacting protein kinase 3 (RIP3).^14, 15^ These two kinases are integral to the necrosome and regulate its function.^14, 19, 20, 21^ Activation of the necrosome increases permeability of the plasma membrane with the subsequent leakage of intracellular contents. This in turn activates an inflammatory ‘danger' or ‘damage' signal thus accounting for necrosis-associated inflammation.^16^ There is increasing evidence that necroptosis may contribute to neuronal death in neurodegenerative disorders, including Gaucher disease,^22^ Huntington disease,^23^ and amyotrophic lateral sclerosis.^24^ The signalling cascades leading to the establishment of the necrosome can be inhibited by small molecules, including necrostatin-1 (Nec1), an allosteric RIP1 inhibitor.^20^ Treatment with Nec1 was reported to be neuroprotective in NMDA-mediated excitotoxicity^25^ and cerebral ischemia.^26^ Furthermore, it has been shown to be efficacious to target the necroptosis pathway in mouse models of Huntington disease^23^ and amyotrophic lateral sclerosis.^24^ In this paper, we investigate the role of necroptosis in NPC1 and evaluate the potential of inhibiting necroptosis as a therapy for NPC1.

## Results

### Decreased viability in NPC1-mutant fibroblasts

**Figure 1:**
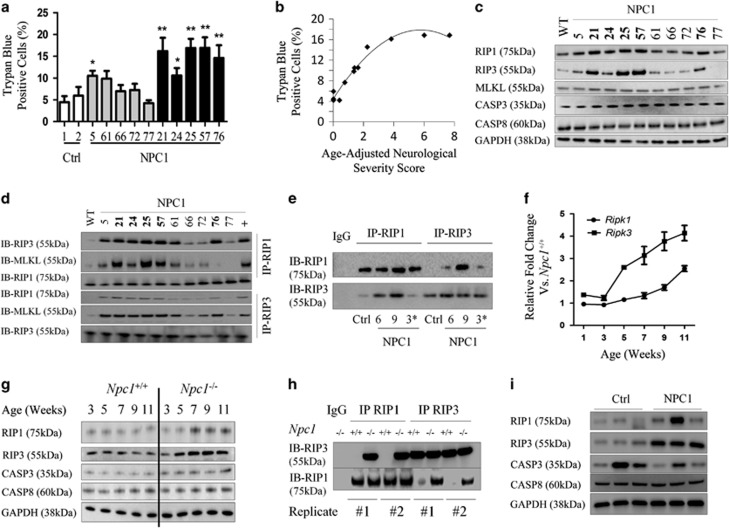
Activation of the necroptotic pathway in NPC1. (**a**) Viability of skin fibroblasts grown in standard culture media was determined by measuring the fraction of trypan blue-positive cells using a TC10 automated cell counter. Increased spontaneous death was observed in NPC1 cell lines compared with controls. White bars indicate controls. Grey and black bars correspond to cell lines from subjects with AANSS scores < and >1.5, respectively. Statistical comparison is relative to the control lines. *P<0.05, **P<0.01 Mann–Whitney, *U*-test. (**b**) Spontaneous cell death in NPC1 fibroblast lines increases with increasing neurological phenotypic severity manifested by the subject from whom the cell line was derived. (**c**) Western blotting analysis of fibroblast lysates to detect expression of RIP1, RIP3, MLKL, Caspase 3, Caspase 8 and glyceraldehyde 3-phosphate dehydrogenase (GAPDH). NPC1 cell lines in bold are derived from a subject with an AANSS>1.5. (**d**) Immunoblot (IB) analysis of RIP1, RIP3 and MLKL after IP with either anti-RIP1 (IP-RIP1) or anti-RIP3 (IP-RIP3). NPC1 cell lines with AANSS>1.5 are indicated by bold typeface. (**e**) RIP1 and RIP3 expression evaluated by western blotting after IP with anti-RIP1 or anti-RIP3 antibodies performed on lysates from control and NPC1 iPS cell-derived neuronal progenitor cells. The iPS cell lines in this figure are derived from GM0565 (Ctrl), NPC6 (6), NPC9 (9), and GM03123 (3*). (**f**) Time course of mRNA expression of *Ripk1* (circle) and *Ripk3* (square) from 1 to 11 weeks in *Npc1*^−/−^ mouse cerebellar brain tissue relative to expression in age-matched *Npc1*^+/+^ mice (*n*=6). Data are shown as mean±S.E.M. (**g**) Time course of protein expression in cerebellar brain tissue from *Npc1*^*+/+*^ and *Npc1*^−/−^ mice evaluated by western blotting analysis. Expression of RIP1, RIP3, caspase 3, and caspase 8 from 3 to 11 weeks was characterized. Experiments were repeated on six independent animals. GAPDH was used as a loading control. (**h**) Immunoblot (IB) analysis of RIP1 and RIP3 immunoprecipitated with either anti-RIP1 (IP RIP1) or anti-RIP3 (IP RIP3) on mouse cerebellar lysate (*n*=6). IP with nonspecific IgG serves as a negative control. Results of two separate IPs (#1 and #2) are shown. (**i**) RIP1, RIP3, caspase 3, and caspase 8 expression evaluated by western blotting in age-matched patient cerebellar lysate

We determined the *in vitro* viability of fibroblasts derived from well-characterized NPC1 subjects being followed in a natural history trial at the National Institutes of Health (NCT00344331), using a panel of NPC1 fibroblasts representing the NPC1 phenotypic spectrum (Table 1). Age-adjusted neurological severity scores (AANSSs) ranged from 0 to 7.7 using the NIH Neurological Severity Score.^27^ When cultured in standard conditions and medium (DMEM plus 10% FBS), NPC1 fibroblast lines typically demonstrated an increased percentage of trypan blue-stained cells (range: 4.25–16.9%) in comparison to control lines (range: 4.5–6%, Figure 1a). Mean percentage of trypan blue-stained cells for two control lines was 5.2±1.2. In contrast, fibroblasts from subjects with an AANSS<1.5 had a mean value of 7.8±0.6 (*n*=5, *P*<0.001) and from subjects with an AANSS>1.5 had a mean value of 15.6±1.1 (*n*=5, *P*<0.001). The relationship between trypan blue-positive cells and AANSS is shown in Figure 1b. The percentage of trypan blue-positive cells initially increases with AANSS and then plateaus above an AANSS of 4. The increased rate of death in NPC1 fibroblasts was confirmed by measuring release of lactate dehydrogenase (LDH) into the medium and by SYTOX Green staining (Supplementary Figures S1a and b). As observed with trypan blue staining, both increased LDH release and SYTOX Green staining were increased in NPC1 fibroblasts from subjects with AANSS>1.5. Mean levels of LDH activity in the culture medium were 7.3±1.3, 9.2±1 (*P*=0.05 *versus* control), and 25.0±2.3 (*P*<0.001 *versus* control) Units/l for control, AANSS<1.5, and AANSS>1.5 lines, respectively. Mean fraction of SYTOX Green positive cells were 1.1±0.2%, 8.8±0.8% (*P*<0.001 *versus* control), and 20.4±2% (*P*<0.001 *versus* control) for control, AANSS<1.5, and AANSS>1.5 lines respectively.

A series of experiments were performed to exclude apoptosis as the mechanism underlying the increased cell death rate of NPC1 fibroblasts. Control and NPC1 fibroblasts were double stained with Annexin-V and propidium iodide (PI) and analysed by flow cytometry. Annexin-V is an apoptosis marker that labels phosphatidyl serine, and PI labels dead cells with permeabilized plasma membranes. An increase in the fraction of Annexin-V-positive/PI-negative cells is consistent with apoptosis. We observed no difference in the fraction of Annexin-V-positive/PI-negative cells in control (7.0±1.1%), NPC1 AANSS<1.5 (6.4±4.6%), or NPC1 AANSS>1.5 (2.8±0.2%) control samples. Furthermore, using DAPI staining, we did not observe a significant difference in the ratio of condensed nuclei reported to the total number of nuclei per optic field when comparing control (0.8±0.6%), NPC1 AANSS<1.5 (0.2±0.1%), or NPC1 AANSS>1.5 (0.3±0.3%) fibroblast lines. Western blotting analysis demonstrated that neither caspase 3 nor caspase 8 protein levels were increased in NPC1 fibroblasts (Figure 1c). These data argue against increased apoptosis as the mechanism underlying the increased cellular death observed in NPC1 fibroblasts.

We next explored whether increased necroptosis was observed in cultured NPC1 fibroblasts. Consistent with necroptosis, western blotting analysis demonstrated increased expression of RIP1 and RIP3 protein in NPC1 fibroblasts compared with control fibroblasts (Figure 1c). Increased protein expression of mixed lineage kinase domain-like (MLKL), a third component of the necrosome complex, was not observed in whole protein lysates. The relationship between relative RIP1, RIP3, MLKL, caspase 3, and caspase 8 average protein relative expression levels and AANSS is shown in Supplementary Figure S2. Only RIP1 levels appear to increase with increased AANSS, and similar to what was observed with trypan blue staining (Figure 1b), RIP1 protein levels plateaued at AANSS values >4.

Necrosome assembly was evaluated by using either anti-RIP1 or anti-RIP3 to immunoprecipitate the necrosomal complex and subsequent probing by western blotting using either anti-RIP1 or anti-RIP3 (Figure 1d). When the necrosomal complex was immunoprecipitated using anti-RIP1, a strong interaction with RIP3 and MLKL was clearly observed in NPC1 fibroblasts. Similarly, when the necrosomal complex was immunoprecipitated using anti-RIP3, increased levels of RIP1 and MLKL were observed in NPC1 fibroblasts compared with control fibroblasts. Of note, the formation of the necrosomal complex appeared to be more prominent in fibroblast lines from subjects with AANSS⩾1.5 (NPC21, 24, 25, 57, and 76). Increased activation of the necroptotic pathway was also observed in neuronal precursors derived from NPC1 iPS cells (Figure 1e). Accumulation of necrosomal proteins was observed in NPC1-derived neuronal stem cells, and consistent with our fibroblast data, immunoprecipitation (IP) followed by western blotting analysis demonstrated increased interaction of RIP1 and RIP3 in NPC1 neuronal stem cells.

### Necroptosis in cerebellar tissue from *Npc1*-mutant mice and NPC1 subjects

We next explored whether increased activation of the necroptotic pathway could be observed in cerebellar tissue from *Npc1*-mutant mice. Gene expression of *Ripk3*, and to a lesser extent *Ripk1*, progressively increased from 3 weeks of age until 11 weeks of age when these mice are typically euthanized owing to significant neurological impairment per Animal Care and Use Committee guidelines (Figure 1f). Western blotting analysis of total protein lysates from control and mutant mouse cerebellar tissue demonstrated increasing RIP1 and RIP3 protein in *Npc1*-mutant mice after 5 and 5 weeks of age, respectively (Figure 1g). No change in the protein levels of either caspase 3 or 8 were observed by western blotting (Figure 1g). Assembly of the necrosomal complex was confirmed by IP of the necrosome using either anti-RIP1 or anti-RIP3 followed by immunoblotting for RIP3 or RIP1, respectively. Increased interaction of RIP1 and RIP3 was observed in cerebellar tissue from 7-week-old *Npc1*^−/−^ mice compared with *Npc1*^+/+^ tissue (Figure 1h).

Western blotting analysis of protein lysates derived from cerebellar tissue from three NPC subjects clearly demonstrated increased protein expression of RIP1 in human brain tissues in two of the three samples and of RIP3 in all three of the samples relative to age-matched control human cerebellar cortex samples (Figure 1i). Consistent with necroptosis and not apoptosis being activated in NPC1 cerebellar tissue, caspase 3 and caspase 8 protein levels were not increased relative to control tissue.

### Inhibition of necroptosis increases NPC1 fibroblast viability

**Figure 2:**
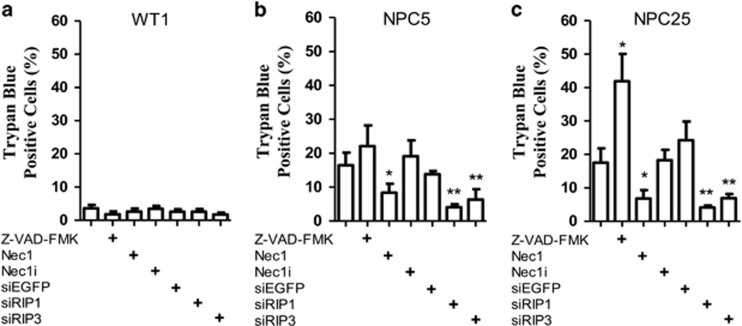
*In vitro* suppression of RIP1 and RIP3. Viability of human skin fibroblasts from control (**a**) and NPC1 (**b** and **c**) subjects was determined by measuring the fraction of trypan blue-positive cells after various treatments to inhibit either apoptosis or necroptosis. For inhibition of apoptosis, the cells were treated with 10 *μ*M of Z-VAD-FMK, a broad caspase inhibitor, for 24 h. For inhibition of necroptosis, the cells were treated with 1 *μ*M Nec1 for 24 h. Treatment with 1 *μ*M Nec1i serves as a negative control. Necroptosis was also inhibited by suppression of RIP1 or RIP3 expression utilizing siRNA. Cells were transfected for 4 h with lipofectamine 2000 complexed with siRIP1 or siRIP3. Treatment of cells with siEGFP served as a negative control. *P<0.05, **P<0.01, Mann–Whitney *U*-test

After establishing necrosomal activation in NPC1, we then evaluated whether inhibition of necroptosis in NPC1 fibroblasts would increase cell viability. No effect on cell viability was observed for control lines when treated with the RIP1 inhibitor, Nec1 (Figure 2a and Supplementary Figure S3). Treatment of NPC1 fibroblasts from subjects NPC5 (AANSS 1.4) and NPC25 (AANSS 6) with 1 *μ*M Nec1 significantly (*P*<0.05) decreased the percentage of trypan blue-stained cells in both NPC1 cell lines (Figures 2b and c). This decrease in trypan blue staining was not observed when the subject cell lines were treated with an inactive analogue of Nec1, Necrostatin-1 inactive (Nec1i). Treatment of NPC1 fibroblasts with Nec1 had no apparent effect on cell morphology or filipin staining (data not shown). Filipin is a fluorescent antibiotic that is used to demonstrate increased lysosomal storage of unesterified cholesterol in NPC1 cells. Suppression of either RIP1 or RIP3 expression utilizing siRNAs resulted in significant suppression of cell death (*P*<0.05) in both NPC5 and NPC25 (Figures 2b and c). Decreased expression of RIP1 and RIP3 protein in siRNA-treated fibroblasts was demonstrated by western blotting (Supplementary Figure S4). Control siRNA targeting either EGFP or the transfection reagents alone did not decrease protein expression (Supplementary Figure S4) or increase either cellular viability or LDH activity (Figure 2, Supplementary Figures S3 and S5). NPC1 cell lines from patients of differing severity demonstrated a similar response to Nec1 inhibitor as that of NPC5 and NPC25, with a higher increased survival observed in cell lines obtained from subjects with a more severe NPC1 phenotype (Supplementary Figures S3 and S5). Efficacy of Nec1 was also evaluated in fibroblast cultures treated with TNFα to induce necroptosis. When evaluating the percentage of trypan blue-positive cells, Nec1 reduced the cytotoxic effects of TNFα from 67.3±4.1 to 25.9±6.1%, 44.3±3.5 to 18.7±3.4%, and 56.0±5.9 to 16.5±2.9% for control (*P*<0.01), AANSS<1.5 (*P*<0.01), and AANSS>1.5 (*P*<0.01) lines, respectively (data not shown). These data were confirmed by measuring LDH release into the medium (Supplementary Figure S5).

As a control, we also evaluated the *in vitro* effects of inhibition of apoptosis using Z-VAD-FMK, a broad caspase inhibitor. No effect on cell viability was observed for control lines (Figure 2a and Supplementary Figure S3); however, exposure of NPC25 cells to Z-VAD-FMK actually decreased cellular viability in NPC25 (Figure 2, *P*<0.05). This later observation is consistent with the known crosstalk between caspase-dependent and -independent cellular death and the role of caspases in suppressing necroptosis.^13, 14, 15^

### Treatment of *Npc1*^−/−^ mice with Nec1 delays the onset of disease manifestations and prolongs lifespan

**Figure 3:**
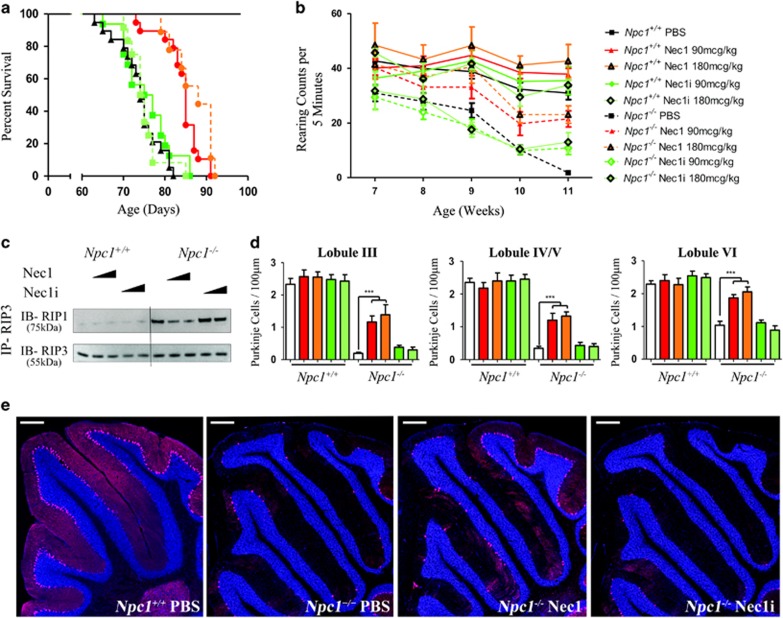
Inhibition of RIP1 decreases NPC1 pathology and increases lifespan in *Npc1*-mutant mice. (**a**) Kaplan–Meier curve demonstrating increased survival of *Npc1*^−/−^ mice treated with either 90 or 180 mcg/kg of Nec1 by i.p. injection every 2 days starting at 3 weeks of age. Mean survival increased from 73.8±1.3 days in vehicle-treated mice (*n*=10) to either 83.2±1.5 (*n*=10, *P*<0.001) or 86.9±1.4 (*n*=10, *P*<0.001) days for mice dosed with either 90 or 180 mcg/kg, respectively. No increase in survival was observed when mice were treated with Nec1i, an inactive analogue of Nec1. (**b**) Total rearing counts were quantified on a weekly basis between 7 and 11 weeks of age in mice treated with PBS (vehicle), Nec1i (90 and 180 mcg/kg), or Nec1 (90 and 180 mcg/kg). Mice treated with Nec1 demonstrate a delay in the progression of neurological symptoms. Data are expressed as the mean±S.E.M., *n*=10/group. (**c**) Immunoblot analysis of RIP1 and RIP3 immunoprecipitated from cerebellar lysates from 7-week-old mice treated with either 90 or 180 mcg/kg of Nec1 or Nec1i. (**d**) Purkinje cell density was quantified in cerebellar lobules III, IV/V, and VI from 7-week-old animals that had been treated for 4 weeks. Purkinje cell loss typically proceeds in a rostral to caudal direction with preservation of lobule X. *Npc1*^*+/+*^ and *Npc1*^−/−^ mice were treated with either PBS (white), 90 mcg/kg Nec1 (red), 180 mcg/kg Nec1 (orange), or Nec1i (green and light green). ***P<0.001, Mann–Whitney *U*-test. (**e**) Sagittal cerebellar sections from 7-week-old animals stained with anti-calbindin (red) and DAPI (4,6-diamidino-2-phenylindole; blue). Calbindin is a marker of cerebellar Purkinje cells. The scale bar represents 200 *μ*m

Based on our data demonstrating increased cellular viability in NPC1 fibroblasts treated with Nec1, we tested whether *in vivo* treatment of *Npc1*^−/−^ mice with Nec1 would impact the NPC1 phenotype. *Npc1*^*+/+*^ and *Npc1*^−/−^ mice were treated with either Nec1, Nec1i, or vehicle (phosphate-buffered saline (PBS)) administered every 2 days by intraperitoneal (i.p.) injection initiated after weaning at 3 weeks of age. Nec1 and Nec1i were studied at 90 and 180 mcg/kg. Control mice did not demonstrate any adverse drug-related physiological or behavioural side effects at either dose level. Relative to vehicle-treated *Npc1*^−/−^ mice, lifespan was increased in *Npc1*-mutant mice by an average of 10±4 (*P*<0.05) and 13±4 days (*P*<0.05) when treated with 90 and 180 mcg/kg of Nec1, respectively (Figure 3a). No survival benefit was observed in *Npc1*-mutant mice treated with Nec1i (*P*=0.72) nor was there a significant difference between the two Nec1 doses (*P*=0.12). Signs consistent with NPC1 disease progression were also delayed in Nec1-treated *Npc1*^−/−^ mice compared with mutant mice treated with either vehicle or Nec1i. Figure 3b and Supplementary Figure S6a demonstrate improved rearing in Nec1-treated *Npc1*^−/−^ mice with an approximate 2-week delay in the worsening of motor function. Compared with *Npc1*^+/+^ mice, a significant decrease (*P*<0.05) in rearing activity was observed after 8 weeks of age in PBS- and Nec1i-treated *Npc1*^−/−^ mice. In contrast, a significant decrease in rearing activity was not observed until 10 weeks of age in Nec1-treated *Npc1*^−/−^ mice (Supplementary Figure S6a). IP of the necrosome using anti-RIP3 demonstrated decreased interaction with RIP1 protein in the cerebellum from *Npc1*^−/−^ mice treated with Nec1 but not from mice treated with Nec1i (Figure 3c).

A pathological hallmark of NPC1 disease is the progressive loss of cerebellar Purkinje cells. This loss of cerebellar Purkinje cells occurs in a stereotypic manner, progressing from anterior to posterior lobules.^28^ Histological evaluation at 7 weeks of age demonstrated preservation of anterior lobule cerebellar Purkinje cells in Nec1 (90 mcg/kg) treated *Npc1*^−/−^ mice compared with either vehicle or Nec1i (90 mcg/kg) treated *Npc1*^−/−^ mice (Figures 3d and e and Supplementary Figure S6b). This visual comparison was quantified by measuring cerebellar Purkinje cell density. In 7-week-old *Npc1*^−/−^ mice, cerebellar Purkinje cell counts were significantly increased in lobules I–IX in Nec1 (90 and 180 mcg/kg) treated *Npc1*^−/−^ mice compared with either vehicle or Nec1i (90 and 180 mcg/kg) treated *Npc1*^−/−^ mice. No significant increase was measured between animals treated with 90 and 180 mcg/kg, suggesting that the maximal efficacy of Nec1 was already achieved at 90 mcg/kg (Figures 3d and e). In contrast, histological evaluation of sagittal cerebellar sections at the time of euthanasia (which varied for the treatment groups) demonstrated no appreciable difference between animals treated with vehicle, 90 mcg/kg Nec1, or 90 mcg/kg Nec1i (Supplementary Figures S7 and S8). In all three cases, there was preservation of lobule X calbindin D-positive cerebellar Purkinje cells but absence of cerebellar Purkinje cells in the anterior lobules. These data are consistent with a delay, but not prevention, of cerebellar Purkinje cell loss in *Npc1*-mutant mice.

## Discussion

NPC1 is a lysosomal storage disorder characterized by the endolysosomal storage of unesterified cholesterol and other lipids, such as sphingosine and glycosphingolipids.^1, 29^ The progressive neurological manifestations of NC1 are ultimately due to loss of neurons. In particular, the ataxia, which is a prominent neurological symptom of the disorder, is due to loss of cerebellar Purkinje cells. The cascade of events precipitated by the endolysosomal accumulation of unesterified cholesterol and lipids has not been fully delineated. Prior work has demonstrated that neuroinflammation,^30^ mitochondrial dysfunction,^31^ impaired axonal transport,^32^ impaired lysosomal calcium homeostasis,^4^ and oxidative stress^31, 33^ are likely contributors to the pathological cascade that ultimately results in neuronal loss. In this paper, via a series of *in vitro* and *in vivo* experiments, we demonstrate that activation of the necroptotic pathway contributes to neuronal death in NPC1 and that inhibition of RIP1 function may have therapeutic benefit.

Necroptosis is a specific mechanism of programmed cell death that has been shown to be associated with neurodegenerative disorders^21^ and with Gaucher disease,^22^ a lysosomal storage disorder like NPC1. Necroptosis is initiated by formation of the necrosome, a multiprotein complex containing RIP1 and RIP3 kinases.^14^ In addition to being a regulator of the necrosome, RIP1 regulates inflammatory signalling initiated by activation of TNFα and Toll-like receptors.^16^ Notably, necroptosis appears to be a cellular death mechanism present in neurodegenerative disorders with neuroinflammation.^18, 23, 24^ Neuroinflammation has been described in NPC1 and has been postulated to contribute to NPC1 disease progression,^30, 34, 35^ and prior pathological studies reporting necrosis suggested that necrosis was contributing to neuronal death in NPC1 brain tissue.^36^ In this manuscript, we utilized a series of *in vitro* experiments to demonstrate activation of necroptosis in NPC1 fibroblasts and iPS cell-derived neuronal precursors. Of note, activation of the necroptotic pathway positively correlates with disease severity. We also demonstrated that the necroptotic pathway is activated in both NPC1-mutant mouse and human patient brain tissue, thus strongly suggesting that necroptosis has a pathological role in NPC1. RIP1 is the major regulator of necroptosis^20^ and its activity can be inhibited by Nec1. Inhibition of RIP1 by Nec1 is effective in prolonging cell viability, decreasing cellular spontaneous death in NPC1 both *in vitro* and *in vivo*. Treatment of *Npc1*^−/−^ mice with Nec1 resulted in delayed cerebellar Purkinje cell loss, delayed progression of neurological manifestations, and significantly prolonged lifespan. These findings were not observed when *Npc1*^−/−^ mice were treated with vehicle alone or Nec1i, an inactive variant of Nec1. Lifespan increased from 73.8±1.3 days to 83.2±1.5 (13%) and 86.9±1.4 (18%) days in mice treated with 90 and 180 mcg/kg of Nec1, respectively. Although significant, the survival benefit we observed in the NPC1 mouse model with Nec1 is less than that observed with either miglustat^37^ or 2-hydroxypropyl-*β*-cyclodextrin.^38, 39^ We suspect that this modest increase in lifespan may be due to sub-optimal pharmacokinetic properties of Nec1 and the dosing frequency used in this study. Nec1 has a half-life of approximately 1 h,^40^ and i.p. injections every 2 days, although practical from the standpoint of animal care, is not optimal. Supporting the contention that Nec1 therapy was suboptimal owing to dosing and pharmacokinetic properties, we observed only a partial decrease of necrosome formation in treated mice (Figure 3c) and observed no significant difference in lifespan when dosing with either 90 or 180 mcg/kg of Nec1. RIP1 inhibitors with better pharmacokinetic properties are being developed,^41, 42, 43, 44^ and these inhibitors will be the subject of further study. In addition to inhibiting RIP1, Nec1 also inhibits indoleamine 2,3-dioxygenase (IDO), which catalyses the rate-limiting step of tryptophan catabolism and an immune regulator.^45^ However, inhibition of IDO unlikely contributes significantly to the observed benefits of Nec1 therapy. The EC50 for inhibition of IDO by Nec1 is approximately 115 *μ*M.^44^ At the high dose of 180 *μ*g/kg, the highest theoretical maximum concentration (Cmax) in blood is 2.25 *μ*g/ml (or 8.7 *μ*M) after IV bolus injection, assuming a blood volume of 80 ml/kg (i.e., 8% body weight). The Cmax after IP injection would be lower than that after IV injection and distribution of the drug beyond the vascular compartment would further lower predicted drug levels. Thus, the likelihood of inhibition of Nec1 on IDO would be remote at the dose levels of 90 and 180 *μ*g/kg used in this study. An alternative mechanism to inhibiting necroptosis via knockdown of RIP1 activity would be inhibition of TNF*α* production. This is an unlikely mechanism in NPC1 as increased production of TNFα has not been observed *in vivo* or *in vivo*.^46^

Targeting the RIP1/RIP3 pathway has been demonstrated to be a potential therapeutic option in other neurodegenerative diseases (e.g., Gaucher,^22^ Huntington^23^) as well as in a rodent stroke models.^47, 48^ In this paper, we demonstrate that necroptosis is involved in the NPC1 pathogenic cascade, and our data indicate that inhibition of RIP1 may have a therapeutic role in treating patients with NPC. Necroptosis is a late, if not the final, step in the NPC1 pathogenic cascade, thus it is not likely that this therapy will be a stand-alone therapy. However, as part of a combined therapy, inhibition of necroptosis in NPC1 may contribute significantly to the treatment of this progressive neurodegenerative disease. RIP1 inhibitors with better pharmacological properties remain to be tested for efficacy in the NPC1 mouse model. In addition, given the clear involvement of the necrosome in NPC1 pathology, inhibition of RIP3 or combined RIP1/RIP3 inhibition should be evaluated for efficacy in NPC1. To our knowledge, this is the first report of the potential benefit of RIP1 inhibition in a lysosomal storage disorder. Work in the Gaucher disease model demonstrated potential efficacy of RIP3 inhibition.^22^ Given that the pathological processes leading to neuronal loss in lysosomal storage disorders are often nonspecific, we postulate that RIP1 or combined RIP1/RIP3 inhibition may also prove effective in significantly delaying neurodegeneration in other lysosomal storage disorders.

## Materials and Methods

### Human subject and animal protections

NPC1 fibroblast cell lines were established as part of a NICHD Institutional Review Board-approved clinical protocol to study the Natural History of NPC (NCT00344331). Guardian permission and, when possible, subject consent/assent were obtained. Animal work was conducted under an approved NICHD Animal Care and Use Committee-approved protocol. Postmortem human cerebellar tissues were obtained from the NICHD Brain and Tissue Bank.

### Fibroblast lines and iPS cells

NPC fibroblast lines were established from skin biopsies. Control lines GM05659C (WT1) and GM03468A (WT2) were obtained from the NIGMS Human Genetic Mutant Cell Repository (Coriell, NJ, USA). Cells were maintained in DMEM (Life Technologies, Carlsbad, CA, USA) containing 10% fetal bovine serum (Omega Scientific, Offenburg, Germany), L-Glutamine (Life Technologies), and penicillin/streptomycin (Life Technologies) and grown on tissue culture plates (BD, San Diego, CA, USA) at 37 °C 5% CO_2_ in a humidified incubator. iPS cells from WT GM05659, NPC6, NPC9, and NPC GM03123 cells were generated, differentiated, and maintained in culture as previously described.^49^

### Cell viability assays

Cell viability was measured using a trypan blue exclusion assay. Cells in suspension were collected and mixed with 0.4% trypan blue solution (Life Technologies) at a 1 : 1 ratio and 10 *μ*l of the cell suspension was then loaded onto the TC10 System (Bio-Rad Laboratories, Hercules, CA, USA) counting slide to quantify the number of viable cells. The leakage of LDH, an intracellular cytoplasmic enzyme, into the medium is a marker of plasma membrane integrity. Loss of plasma membrane integrity is a hallmark of necrosis. Briefly, a reaction mixture of 0.2 mM NADH (Sigma Aldrich, St. Louis, MO, USA) and 0.36 mM sodium pyruvate (Sigma Aldrich) was dissolved in Krebs–Henseleit (K–H) buffer containing 2% bovine serum albumin. The K–H buffer is composed of 118 mM NaCl, 4.8 mM KCl, 1.2 mM MgSO_4_, 1.25 mM CaCl_2_, 1.2 mM KH_2_PO_4_, and 24 mM NaHCO_3_ (pH 7.4). Ten microliters of cell supernatant and 190 *μ*l of reaction mixture were mixed well in 96-well plates prior to spectrophotometric kinetic readings. Owing to the differences in the absorption spectra of NADH and NAD^+^, changes in the NADH concentration can be detected at 340 nm. The decrease in absorbance measured every minute over a 10-min period represents the activity of LDH. One unit of LDH activity is defined as the quantity for oxidation of 1 *μ*mol NADH per minute; the LDH activity of cell supernatant was expressed in units per liter (Unit/l). We also monitored loss of plasma membrane integrity by measuring SYTOX Green (Molecular Probes–Invitrogen, Eugene, OR, USA) Hoechst 3342 (Molecular Probes) double staining by immunofluorescence.

### Apoptosis assay

To examine the percentage of apoptotic cells, the measurements were carried out using FACS Calibur (BD, San Diego, CA, USA) or fluorescence microscopy. Cells were trypsinized and stained in solution with Annexin V-fluorescein and PI for 20 min at 25 °C in the dark using the FITC Annexin-V apoptosis Detection Kit I (BD). After washing with ice-cold PBS, fluorescence was measured by flow cytometry. For assessment of apoptosis by fluorescence microscopy, fibroblasts were stained with DAPI and mounted on glass slides using Mowiol (Calbiochem, San Diego, CA, USA). For each experiment and each condition, at least 200 cells were scored in a blinded manner by counting the percentage of condensed nuclei in five random fields for each condition.

### Animal breeding, treatment and tissue collection

*Npc1*^*+/−*^ mice were intercrossed to obtain *Npc1*^*+/+*^ and *Npc1*^*−pc*^ mice. Pups were weaned 3 weeks after birth; water and normal mouse chow were available *ad libitum*. Genotyping PCR was performed using tail DNA.^50^ Mice were euthanized at 1, 3, 5, 7, 9, and 11 weeks of age. Cerebellum were collected from both mutant and control animals and immediately frozen on dry ice. *Npc1*^*+/+*^ and *Npc1*^−/−^ mice were randomly placed in one of the treatment groups: Vehicle (PBS), Nec1 (PubChem CID 2828334; Millipore, Billerica, MA, USA), or Nec1i (PubChem CID 5371761; Millipore). Treatment was initiated at 3 weeks. Mice were treated every 2 days by i.p. injection with PBS or 90 mcg/kg Nec1, 180 mcg/kg Nec1, 90 mcg/kg Nec1i, or 180 mcg/kg Nec1i. After 4 weeks of treatment, 10 animals per group were euthanized and the other 10 were euthanized when survival end point criteria were reached, which were: hunched posture and reluctance to move about the cage, inability to remain upright when moving forward, and weight loss >30% of peak weight. Age-matched *Npc1*^+/+^-injected animals were euthanized for comparison. Mice were euthanized in a CO_2_ chamber and then transcardially perfused with PBS for biochemical and molecular analysis or transcardially perfused with 4% paraformaldehyde solution to fix the tissue prior to pathological analysis.

### Immunohistochemistry

Brain cerebellar tissue was processed as described.^34^ Mice were killed at 7 weeks by CO_2_ asphyxiation and transcardially perfused with ice-cold 4% paraformaldehyde in PBS, pH 7.4. The brains were postfixed in 4% paraformaldehyde solution for 24 h and then cryoprotected in 30% sucrose. Cerebellar tissues were cryostat-sectioned parasagittally (20 mcm) and floating sections were collected in PBS with 0.25% Triton × 100. Sections were incubated overnight at 4 °C with rabbit anti-calbindin (1 : 2000, Swant, Marly, Switzerland) and DAPI (1 : 5000, Invitrogen, Carlsbad, CA, USA), detected using Dy-Light-594 goat anti-mouse IgG (Vector, Burlingame, CA, USA).

### RNA extraction

Total RNA was extracted from mouse cerebellum tissues, using TRIzol reagent (Life Technologies), followed by purification with Qiagen RNeasy Mini Columns (Qiagen, Hilden, Germany). For gene expression analysis by real-time qPCR, the quality and the quantity of RNA were assessed using NanoDrop (Thermo Scientific, Inc., Pittsburgh, PA, USA). One mcg of total RNA was reverse-transcribed to obtain cDNA using a High-Capacity cDNA Archive Kit, according to the manufacturer's instructions (Life Technologies). The following TaqMan assays were used to assess the relative expression level of: *Ripk1* (Mm00436354_m1), *Ripk3* (Mm01319233_g1), and *Gapdh* (Mm03302249_g1). Amplifications were performed in 96-well plates with an Applied Biosystems 7300 real-time PCR system (Carlsbad, CA USA). Each sample was analysed in triplicate, using 50 ng of total cDNA for each reaction, as described.^51^

### Protein extraction and western blottings

Total proteins were extracted from cells, patient samples and mouse tissues using RIPA buffer (50 mM Tris-HCl, pH7.4, 1% NP-40, 0.5% sodium deoxycholate, 0.1% SDS, 150 mM NaCl, 2 nM EDTA, 40 mM NaF, 0.2 mM Na_3_VO_4_) with complete protease inhibitor cocktail (Roche, Basel, Switzerland). Protein amounts were determined using the BCA assay (Bio-Rad Laboratories). Protein lysates (typically 5–20 *μ*g) were loaded onto a 4–12% gradient NuPAGE Gel (Invitrogen, Carlsbad, CA, USA), and electrophoresis was carried out at a constant 120 V. Protein transfer was performed using the iBLOT dry transfer setup (Life Technologies) according to the manufacturer's protocol. Following transfer, nitrocellulose membranes were incubated in a 2% BSA and PBS 0.1% Tween 20 blocking buffer for 1 h at room temperature, followed by incubation with the primary antibody at 4 °C overnight. After the initial incubation, the membrane was rinsed and incubated with the appropriate secondary antibody for 1 h at room temperature. Membrane development was carried out using the Bio-Rad Chemiluminescence Detection Kit (Hercules, CA, USA). Primary antibodies used include: hRIP3 (1 : 1000, Abcam, Cambridge, UK), mRIP3 (1 : 1000, Sigma Aldrich), RIP1 (1 : 800, Cell Signaling Technology, Danvers, MA, USA), MLKL (1 : 1000, Abcam), Caspase 3 (1 : 2000, Cell Signaling Technology), caspase 8 (1 : 2000, Cell Signaling Technology), GAPDH (1 : 2000, Cell Signaling Technology), anti-mouse IgG HRP, or anti-rabbit IgG HRP (1 : 20000, Sigma Aldrich). For quantitation, band intensities (OD × mm^2^) were normalized to GAPDH using the ImageLab4.1 software (Bio-Rad Laboratories).

### Rearing evaluation

Spontaneous activity of each mouse was recorded weekly until reaching the late humane end point (loss of 1 g body weight within 24 h) as previously described.^34^ Briefly, rearing was recorded manually for 5 min (the number of times the mouse reared on its hind legs with or without support of the cage wall).

### Statistical analysis

Results were presented as mean±S.E.M. Mann–Whitney *U*-test was used to compare between two groups. Differences were considered to be significant with *P-*values of <0.05. Statistical calculations were performed with the GraphPad Prism software (San Diego, CA, USA).

## Figures and Tables

**Table 1:** Primary fibroblast lines and age-adjusted neurological severity score

**Cell line**	**Age-adjusted neurological severity score**
WT1	NA
WT2	NA
GM0565 (iPS)	NA
GM03123 (iPS)	Not available
NPC5	1.4
NPC6 (iPS)	0.5
NPC9 (iPS)	6
NPC21	3.8
NPC24	1.6
NPC25	6
NPC57	7.7
NPC61	1.4
NPC66	0.4
NPC72	0.8
NPC76	2.2
NPC77	0^a^

Abbreviation: NA, not applicable.

a Subject evaluated at <1 year of age.

## Abbreviations

NPC1: Niemann–Pick disease, type C1
IP: immunoprecipitation
RIP1: receptor interacting protein kinase 1
RIP3: receptor interacting protein kinase 3
AANSS: age-adjusted neurological severity score
Nec1: Necrostatin-1
LDH: lactate dehydrogenase
MLKL: mixed lineage kinase domain-like
