# Supplementary material for: Necroptosis in Niemann–Pick disease, type C1: a potential therapeutic target
Source: Cell Death Dis. 2016 Mar 17;7(3):e2147–. doi: 10.1038/cddis.2016.16 (PMC4823930; doi:10.1038/cddis.2016.16)
Supplement: Supplementary Figure Legends [file cddis201616x1.docx]

**Necroptosis in Niemann-Pick Disease, type C1: A Potential Therapeutic Target**

SUPPLEMENTAL FIGURE LEGENDS

**Suppl. Fig.1** Evaluation of plasma membrane integrity. (a) Control (Ctrl) and NPC1 fibroblasts were grown for 24 hours in normal culture media after which LDH activity in the culture medium supernatant was measured (n=6). (b) Fibroblasts from control and NPC1 subjects were stained with Hoechst 3342 and SYTOX Green for live-cell imaging (n=6). White bars indicate control cell lines, gray bars indicate NPC1 cell lines from subjects with an AANSS<1.5, and black bars indicate cell lines from subjects with an AANSS>1.5. * p<0.05, ** p< 0.01, *** p<0.001, Mann Whitney, U-test *vs*. control cell lines

**Suppl. Fig.2** Correlation between RIP1, RIP3, MLKL, caspase 3 and caspase 8 expression relative to GAPDH and Age Adjusted Neurological Severity Score in control and NPC1 fibroblasts.

**Suppl. Fig.3** Cell viability was determined using trypan blue staining. Cell were incubated for 24 hours, prior staining with 0.4% trypan blue, with Z-VAD-FMK (10µM), Nec1 (1µM), or Nec1i (1µM). Alternatively cells were transfected for 4 hours using lipfectamine 2000 and and small interference RNA targeting either EGFP, RIP1 or RIP3 transcripts (n=6). * p<0.05, ** p<0.01, *** p<0.001, Mann Whitney, U-test *vs*. untreated cell line.

**Suppl. Fig.4** Efficiency of siRNA inhibition was evaluated by western blot analysis of RIP1, RIP3, MLKL and GAPDH protein expression. The depicted experiment was performed using the NPC57 cell line. 2.10^5^ cells were transfected with lipofectamine 2000 with the indicated siRNA (30 nM) for 4 hours.

**Suppl. Fig.5** Determination of cell viability by measuring LDH activity in culture medium supernatant (n=6). Cells were cultured without (white) or with 2.5pg/mL of TNFα (gray), and treated with 1µM Nec1, 1µM Nec1i or 10µM Z-VAD-FMK. * p<0.05, ** p<0.01, *** p<0.001, Mann Whitney, U-test.

**Suppl. Fig.6** (a) Total rearing counts were quantified on a weekly basis between 7 and 11 weeks of age in *Npc1*^-/-^ mice treated with PBS (vehicle), Nec-1i (90 and 180 mcg/kg) or Nec-1 (90 and 180 mcg/kg). Mice treated with Nec-1 demonstrated a delay in the progression of neurological manifestation (n=10/group. *Npc1*^+/+^ Mice injected with PBS (black), *Npc1*^-/-^ Mice injected with PBS (white), Necrostatin-1 90mcg/kg (red) or 180mcg/kg (orange) or Necrostatin-1 inactive control 90mcg/kg (green) or 180mcg/kg (light green). (b) Analysis of Purkinje cell density in the indicated cerebellar lobule after 4 weeks of treatment. n=10/group, * p<0.05, ** p<0.01, *** p<0.001, Mann Whitney, U-test. Data is expressed as the mean ± SEM. ** p<0.01, Mann Whitney, U-test vs. *Npc1*^+/+^. Mice (n=10/group) injected with PBS (white), Necrostatin-1 90mcg/kg (red) or 180mcg/kg (orange) or Necrostatin-1 inactive control 90mcg/kg (green) or 180mcg/kg (light green).

**Suppl. Fig.7** Terminal-stage analysis of cerebellar Purkinje cell density. Purkinje cell density was evaluated in the indicated lobules. Mice (n=10/group) injected with PBS (white), Necrostatin-1 90 mcg/kg (red) or 180 mcg/kg (orange), Necrostatin-1 inactive control 90 mcg/kg (green) or 180 mcg/kg (light green). No statistical difference (Mann Whitney, U-test) were observed between mice treated with PBS versus mice treated with either Nec1 or Nec1i.

**Suppl. Fig.8** Calbindin immunofluorescence (red) demonstrates the expected anterior-to-posterior cerebellar Purkinje cell loss in midline sagittal sections from *Npc1* mutant mice. For this figure sections were obtained from 15 week old *Npc1*^+/+^ mice (top panels), 7 week old *Npc1^-^*^/-^ treated, as indicated, for 4 weeks (middle panels), and *Npc1^-^*^/-^ mice at the time of euthanasia (end stage, bottom panels). Sections were counterstained with DAPI (blue). Scale bar indicates 200 µm.
