# Supplementary material for: Necroptosis in Niemann–Pick disease, type C1: a potential therapeutic target
Source: Cell Death Dis. 2016 Mar 17;7(3):e2147–. doi: 10.1038/cddis.2016.16 (PMC4823930; doi:10.1038/cddis.2016.16)

Supplemental Figure 1

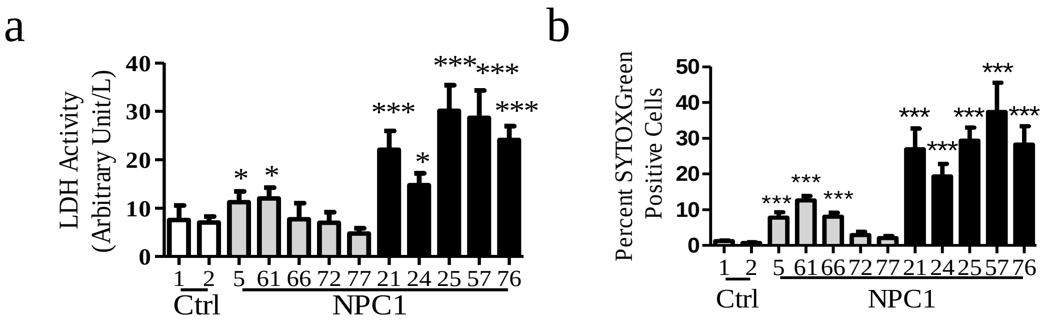

Supplemental Figure 2

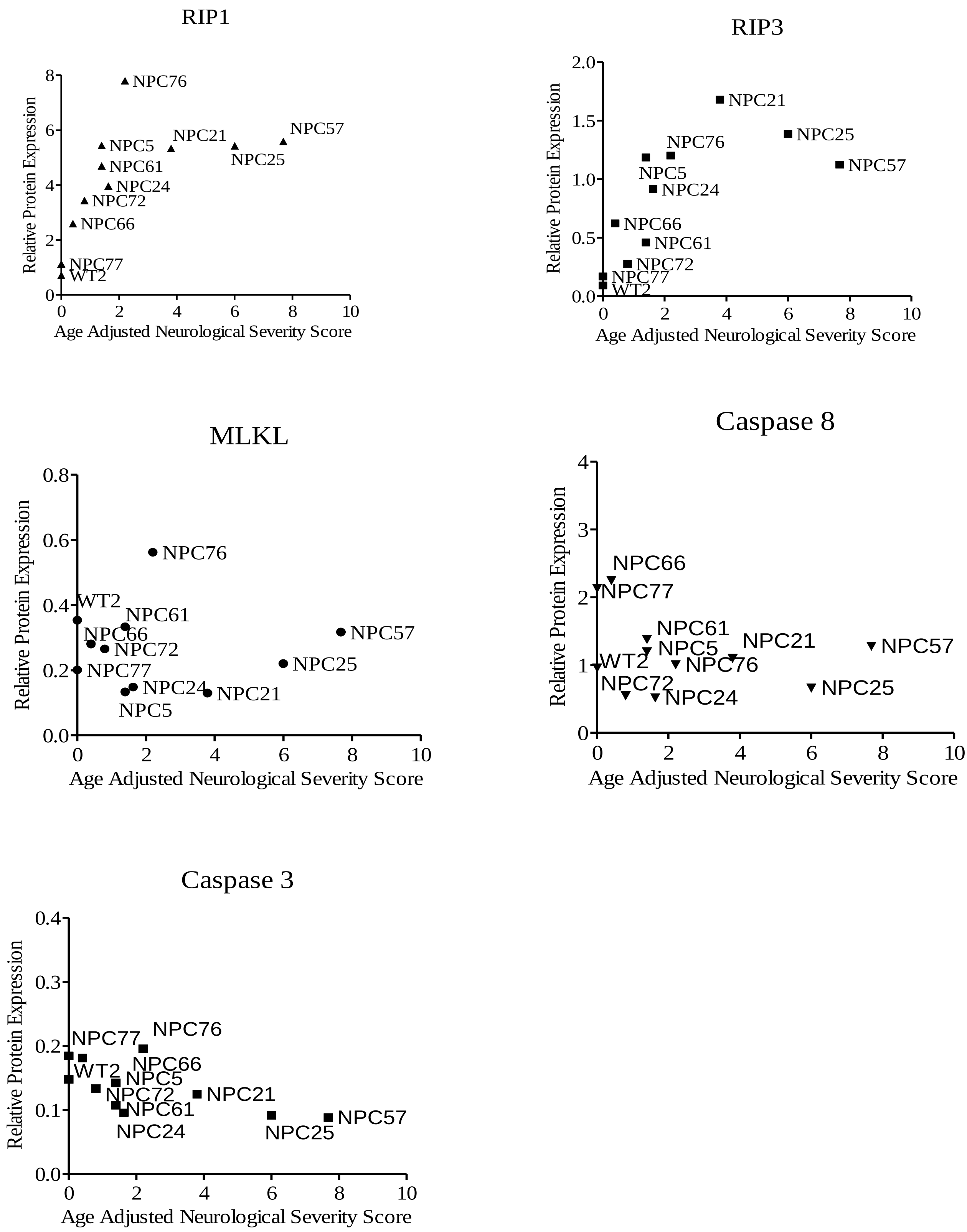

Supplemental Figure 3

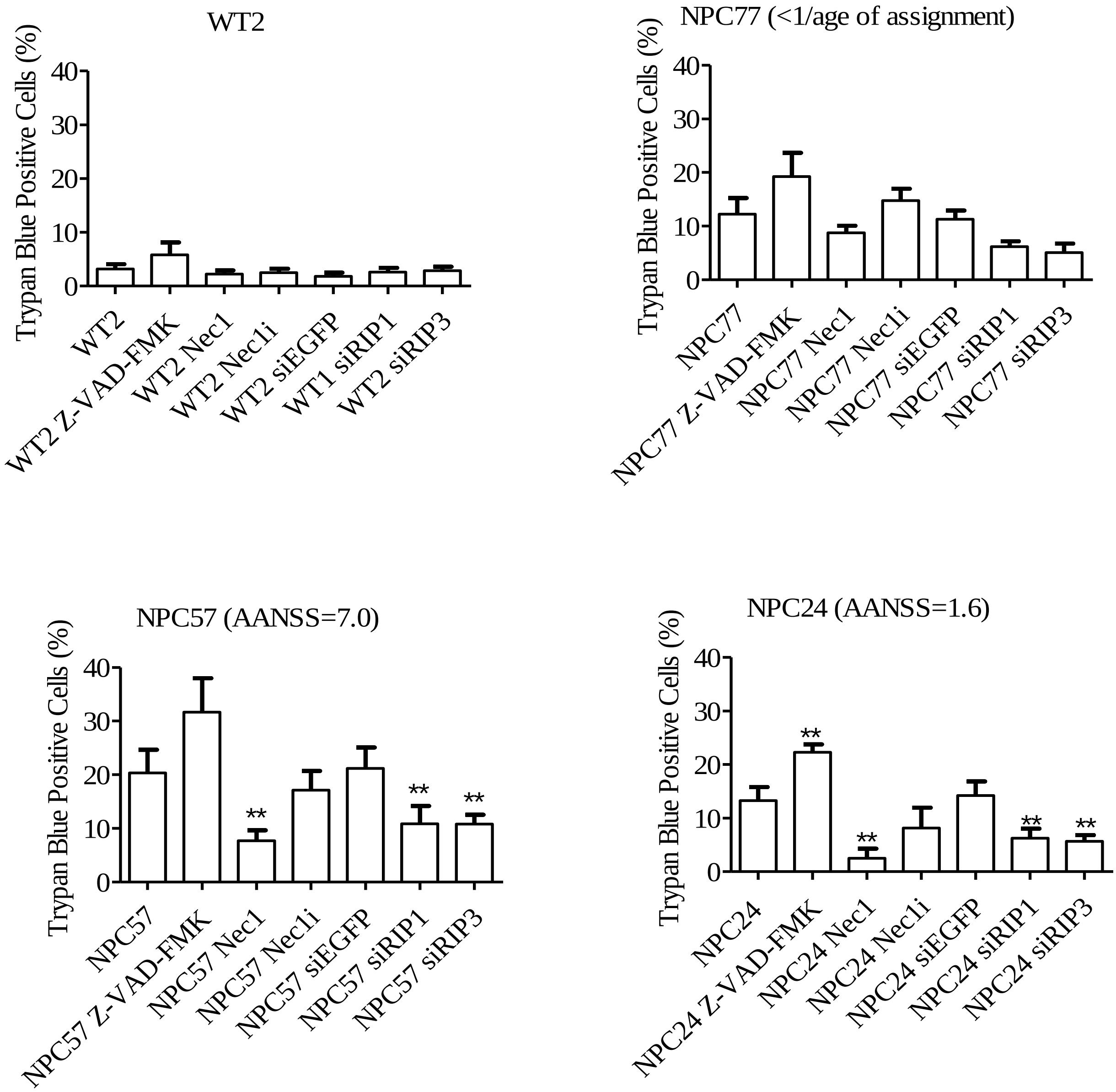

Supplemental Figure 4

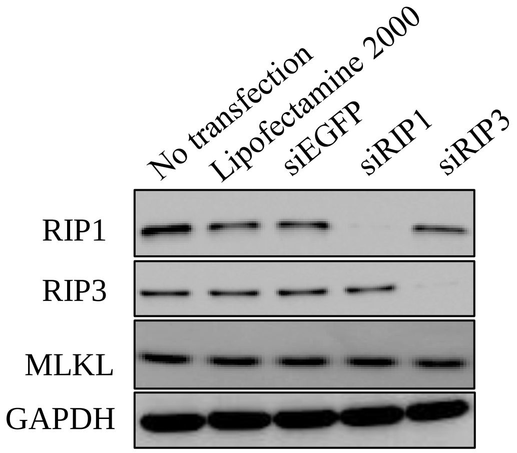

Supplemental Figure 5

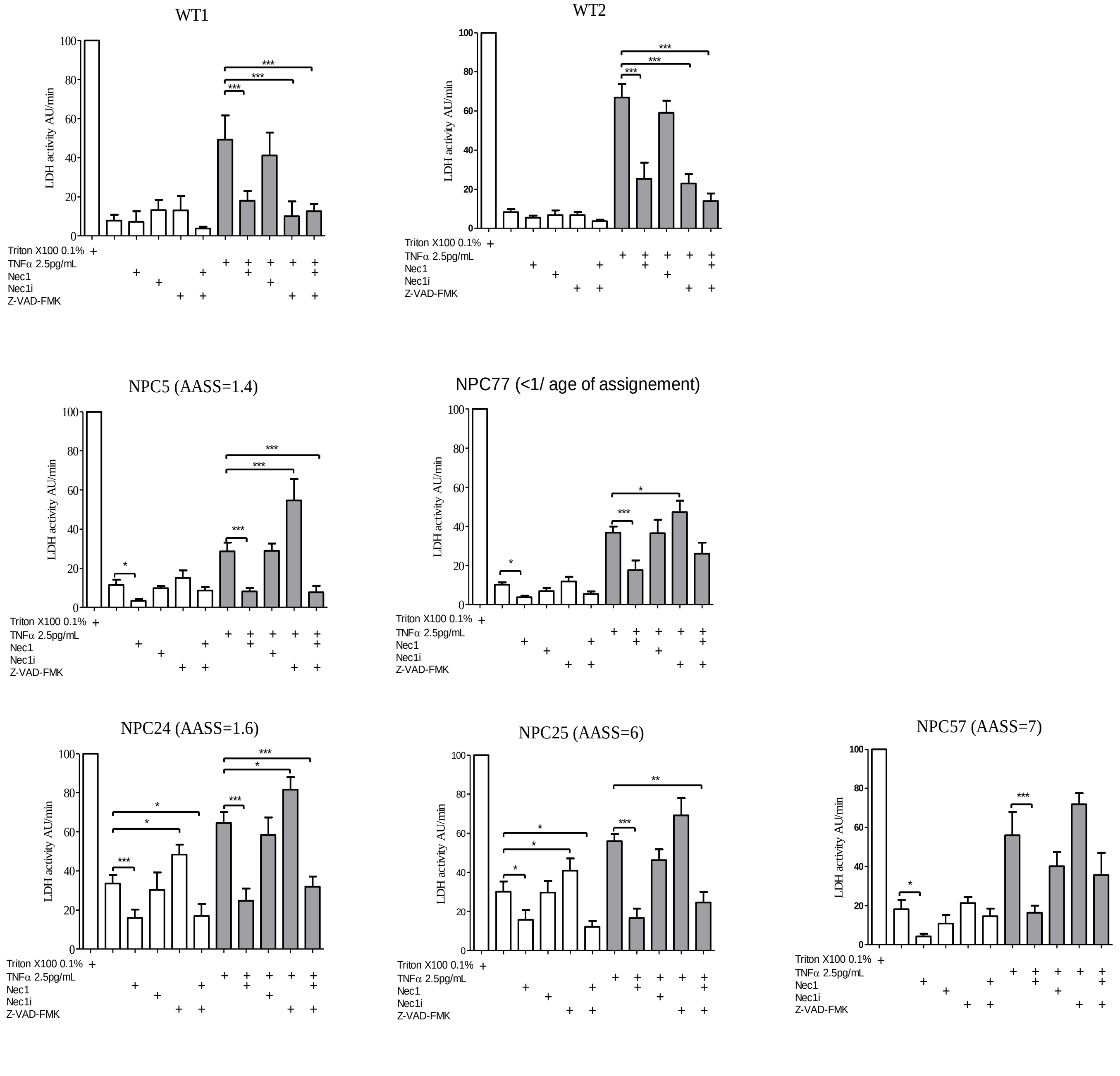

Supplemental Figure 6

a

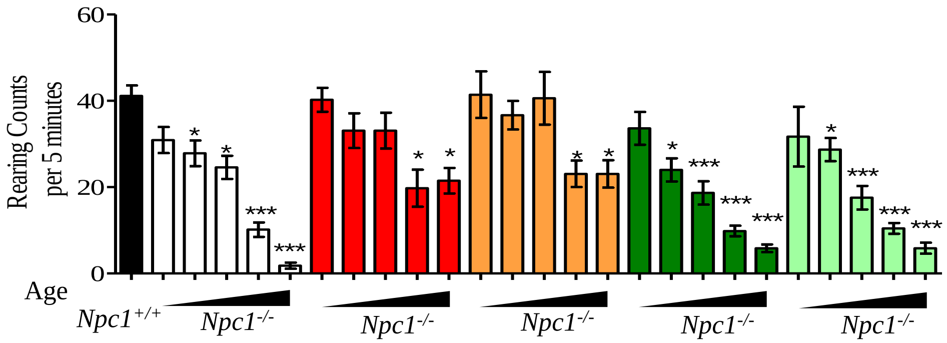

b

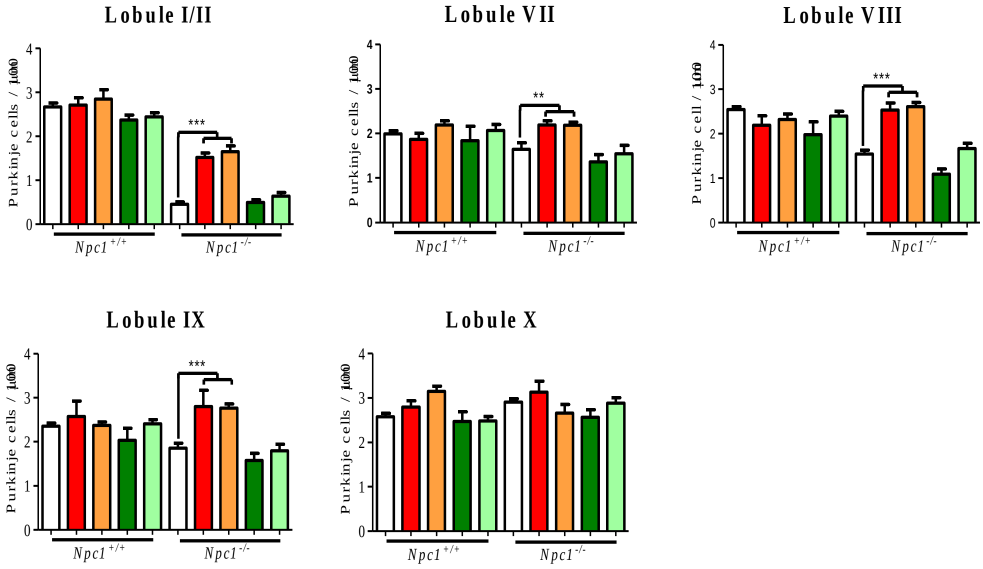

Supplemental Figure 7

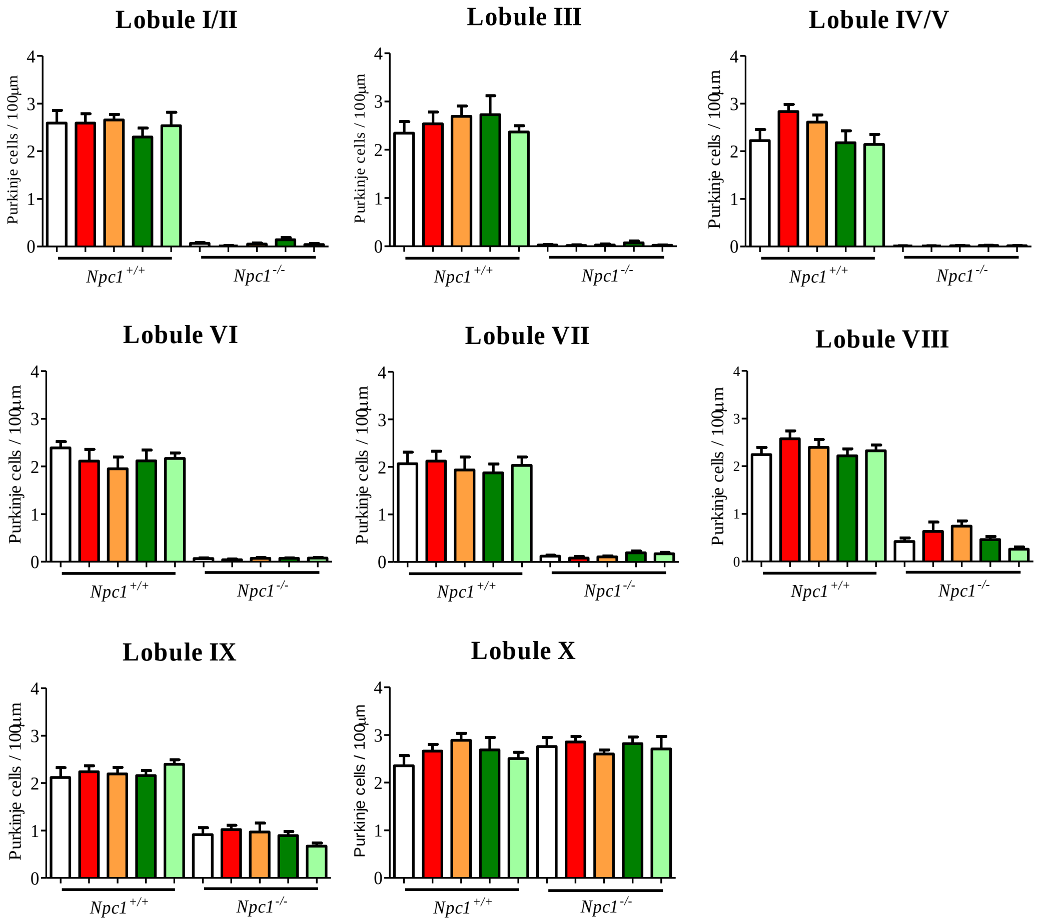

Supplemental Figure 8

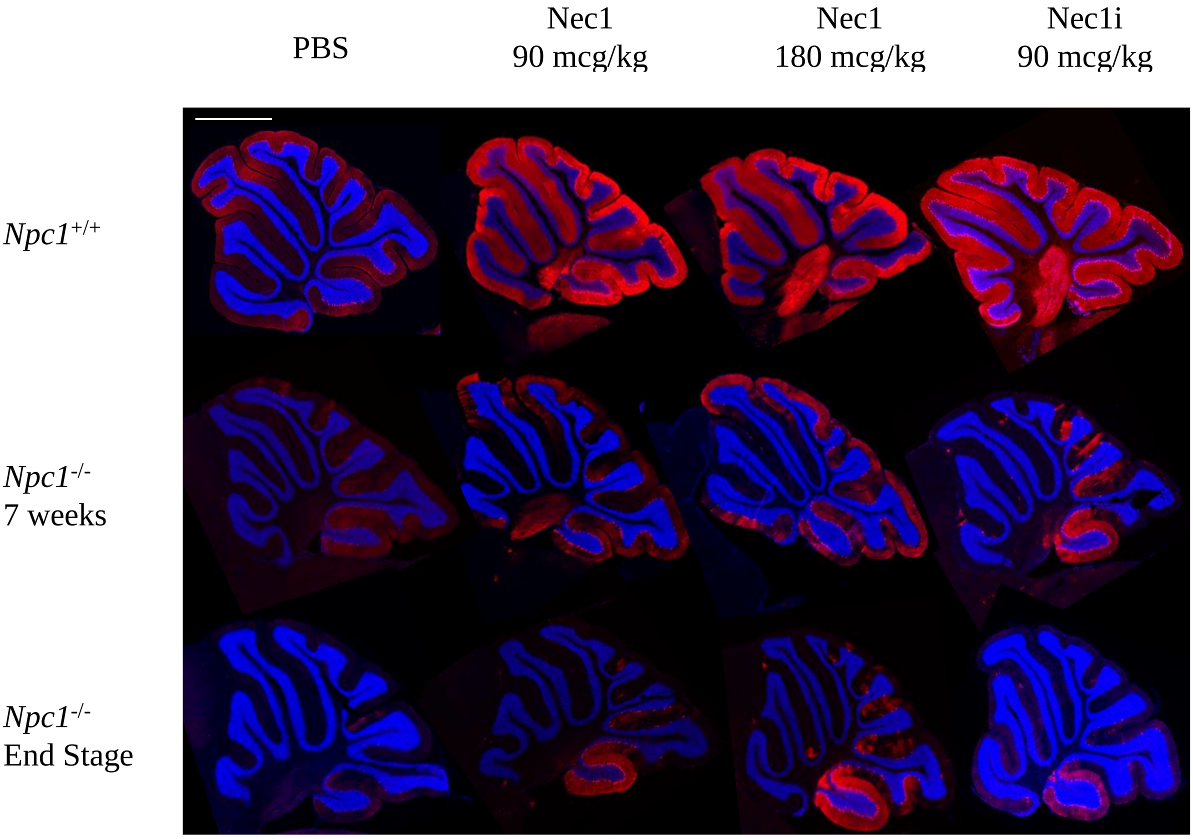

Supplement: Supplementary Figures [file cddis201616x2.pdf]
